# RNA-Seq reveals seven promising candidate genes affecting the proportion of thick egg albumen in layer-type chickens

**DOI:** 10.1038/s41598-017-18389-5

**Published:** 2017-12-22

**Authors:** Yi Wan, Sihua Jin, Chendong Ma, Zhicheng Wang, Qi Fang, Runshen Jiang

**Affiliations:** College of Animal Science and Technology, Anhui Agricultural University, Hefei, 230036 China

## Abstract

Eggs with a much higher proportion of thick albumen are preferred in the layer industry, as they are favoured by consumers. However, the genetic factors affecting the thick egg albumen trait have not been elucidated. Using RNA sequencing, we explored the magnum transcriptome in 9 Rhode Island white layers: four layers with phenotypes of extremely high ratios of thick to thin albumen (high thick albumen, HTA) and five with extremely low ratios (low thick albumen, LTA). A total of 220 genes were differentially expressed, among which 150 genes were up-regulated and 70 were down-regulated in the HTA group compared with the LTA group. Gene Ontology (GO) analysis revealed that the up-regulated genes in HTA were mainly involved in a wide range of regulatory functions. In addition, a large number of these genes were related to glycosphingolipid biosynthesis, focal adhesion, ECM-receptor interactions and cytokine-cytokine receptor interactions. Based on functional analysis, *ST3GAL4*, *FUT4*, *ITGA2*, *SDC3*, *PRLR*, *CDH4* and *GALNT9* were identified as promising candidate genes for thick albumen synthesis and metabolism during egg formation. These results provide new insights into the molecular mechanisms of egg albumen traits and may contribute to future breeding strategies that optimise the proportion of thick egg albumen.

## Introduction

As consumer demand for egg products increases, much attention has been given to the internal quality of eggs. One of the major factors contributing to interior egg quality is the egg white. A fresh, high-quality egg has a firm, gelatinous albumen that anchors the yolk and restricts the growth of microbiological pathogens^1^. Fresh egg albumen consists of three fractions: the outer thin white, the gel known as the thick white and the inner thin white, in addition to the chalazae. The thick egg white is a firm gel, and a large proportion of thick white is desirable, whereas the thin white is a sign of low quality that lead to staleness^2^. Therefore, increasing the percentage of the thick white could be advantageous for poultry production.

The proteins of egg albumen are synthesised and stored in the largest segment of the oviduct, the magnum^3^, and the abundance of proteins in albumen is affected by numerous factors, including genetic characteristics, age and environmental factors^4,5^. The characteristic gel structure of thick egg albumen is thought to be attributed to the ovomucin protein fraction^6^. High-quality eggs contain a significantly higher percentage of thick white and a lower percentage of thin white than lower-quality eggs^7^. For breeding purposes, selection for high and low percentages of thick albumen has been carried out in Rhode Island Reds^8^. The ability of hens to secrete a greater or lesser amount of thick albumen in their eggs was demonstrated to be an inherited, individual characteristic, and layers can be bred to produce eggs with a large amount of thick albumen^9^. However, it has been difficult to develop traditional breeding methods using chicken genetics to maintain high production performance.

With the advent of next-generation sequencing technology^10^, many studies have applied RNA-Seq to analyze various tissues in poultry, including muscle, liver, hypothalamus and ovarian follicle. Researchers have analysed the molecular mechanisms associated with growth rates, carcass traits, heat resistance and feed efficiency^11–15^. However, little attention thus far has been paid to the molecular regulatory mechanisms controlling egg albumen at the transcriptome level. Identifying the genes underlying egg albumen traits and their incorporation into genetic evaluation systems would be valuable for poultry breeding programmes. Some recent progress has been made in the characterisation of genomic regions associated with egg albumen. Two distinct quantitative trait locus (QTL) regions that affect egg white thinning during the production period have been identified on chromosome 2 in chicken^2^, and four QTL regions that affect egg white quality have been found on chromosomes 4, 26, 7 and Z^1^.

The objective of this study was to identify differentially expressed genes (DEGs) in Rhode Island white hens with extremely high or low proportion of thick egg albumen using RNA-Seq. In addition, Gene Ontology (GO) and Kyoto Encyclopedia of Genes and Genomes (KEGG) analyses were carried out to elucidate the biological functions of the differentially expressed genes. This study provides important findings regarding potential regulatory genes and related pathways underlying egg albumen traits, and our results will be instrumental in the use of genetic improvement methods for breeding chickens with high proportions of thick egg albumen.

## Results

### RNA sequencing of chicken magnum tissue

After removing low-quality and adaptor sequences, we acquired a total of 486.44 million clean reads with an average of 54.05 million reads per sample (range: 46.74 to 66.43 million). There was a high percentage of mapped reads, ranging from 82.49 to 86.56%. The Q20 and Q30 quality values were 97.83% and 94.42%, respectively (Supplementary Table S1). Approximately 83.95% of the total reads were uniquely mapped to the *Gallus gallus* genome. Most mapped reads (81.70 to 89.10%) were located within an exon, whereas a smaller percentage of the mapped reads (less than 19.0%) were located within introns and intergenic regions (Supplementary Table S1). Only the uniquely mapped reads were considered in the analysis.

### Differentially expressed genes in the high-thick albumen (HTA) and low-thick albumen (LTA) groups

**Figure 1:**
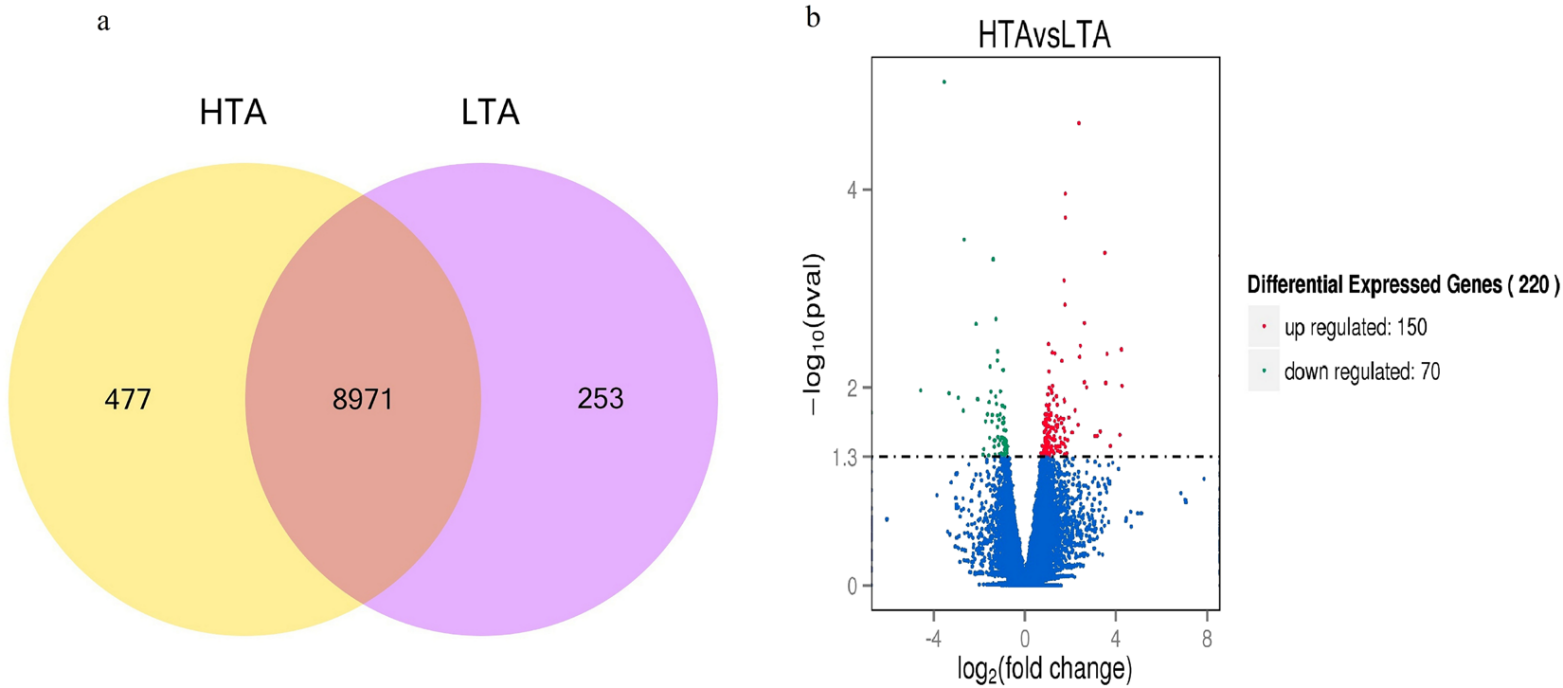
Comparison of gene expression levels and differentially expressed gene distributions between magnum samples from chickens with extremely high (HTA) and low (LTA) thick-to-thin albumen ratios. (**a**) Venn diagram showing genes expressed only in the HTA group (yellow circle), only in the LTA group (light red circle), or common to both groups (intersection). (**b**) Scatter plot of differentially expressed genes (HTA vs. LTA). Red points represent up-regulated genes with a log_2_ (fold change) > 1 and p adj < 0.05. Green points represent down-regulated genes with a log2 (fold change) <−1 and p adj < 0.05. Blue points represent genes showing no significant difference. Fold change = normalised gene expression in the HTA group / normalised gene expression in the LTA group.

**Figure 2:**
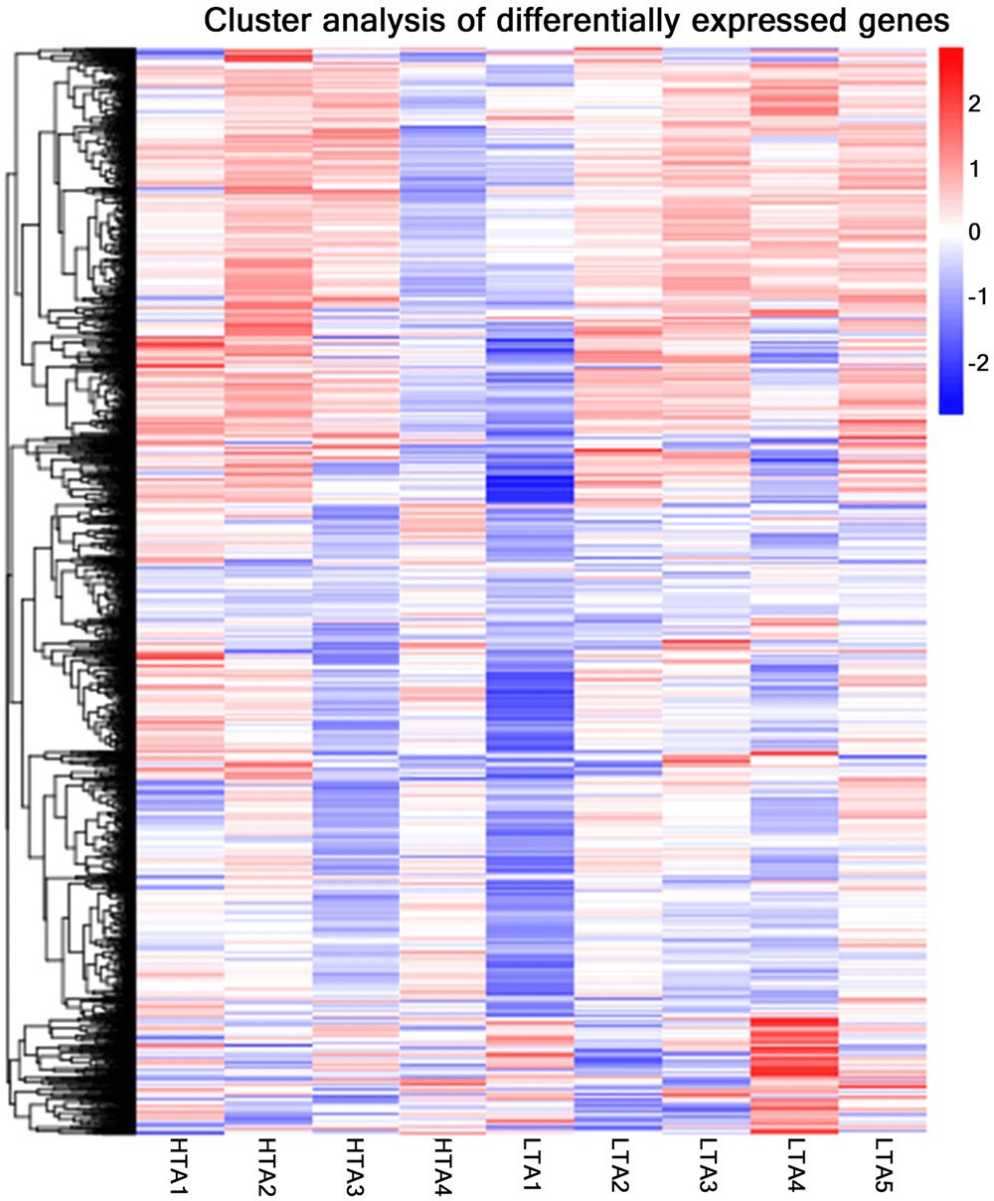
Heatmap of differentially expressed genes. Rows indicate genes showing significant differences in expression between the two groups; columns represent individual samples from the two groups (HTA and LTA represent the high-thick albumen and low-thick albumen groups, respectively).

After mapping to the *G. gallus* genome, 9448 and 9224 genes were obtained from the HTA and LTA libraries, respectively. Among these genes, 477 were expressed only in the HTA group, while 253 were expressed only in the LTA group, and 8971 genes were expressed in both libraries (Fig. 1a). A total of 220 genes were differentially expressed between the two groups, among which 150 were up-regulated, and 70 were down-regulated in the HTA group (Fig. 1b). The details of all the DEGs identified in the comparison of the two groups are shown in Supplementary Table S2. Volcano plots of the genes that were differentially expressed in the two groups illustrate their distinct transcriptional profiles (Fig. 1b). The expression clustered patterns of all DEGs were generated based on the log2 values of their expression ratios (Fig. 2). The top 10 up-regulated and down-regulated genes in the HTA group are listed in Table 1.

**Table 1:** Detailed information about the top 10 up-regulated and down-regulated genes in the HTA group.

Ensembl gene ID	Gene	readcount_HTA	readcount_ LTA	log2 Fold Change	P-value	Up/Down (HTA/LTA)	Gene description
***Up-regulated genes***							
ENSGALG00000019651	*AGR3*	3.53	0.18	4.27	9.60E-03	Up	Anterior gradient homologue3
ENSGALG00000028193	*SEPT4*	4.10	0.22	4.23	4.13E-03	Up	Septin4
ENSGALG00000017169	*ELMOD1*	20.40	1.67	3.61	4.57E-03	Up	ELMO domain-containing 1
ENSGALG00000028532	*FAM178B*	3.20	0.53	3.56	8.99E-03	Up	Family with sequence similarity 178, member B
ENSGALG00000007837	*KAZALD1*	12.66	1.49	3.10	3.09E-02	Up	Kazal-type serine peptidase inhibitor domain 1
***Down-regulated genes***							
ENSGALG00000010929	*SPARCL1*	2.95	12.33	−2.06	1.31E-02	Down	Secreted protein acidic and rich in cysteine-like 1
ENSGALG00000013766	*DSEL*	5.00	21.92	-2.13	2.27E-03	Down	Dermatan sulfate epimerase-like
ENSGALG00000026075	*AMER3*	1.89	12.32	−2.70	1.71E-02	Down	Adenomatous polyposis coli (APC) membrane recruitment 3
ENSGALG00000011041	*DNAJC6*	0.77	7.77	−3.33	1.14E-02	Down	HSP40 homologue subfamily C member 6
ENSGALG00000003446	*PRLR*	96.87	1125.41	−3.54	8.14E-06	Down	Prolactin receptor

### GO and pathway analysis of the DEGs

**Figure 3:**
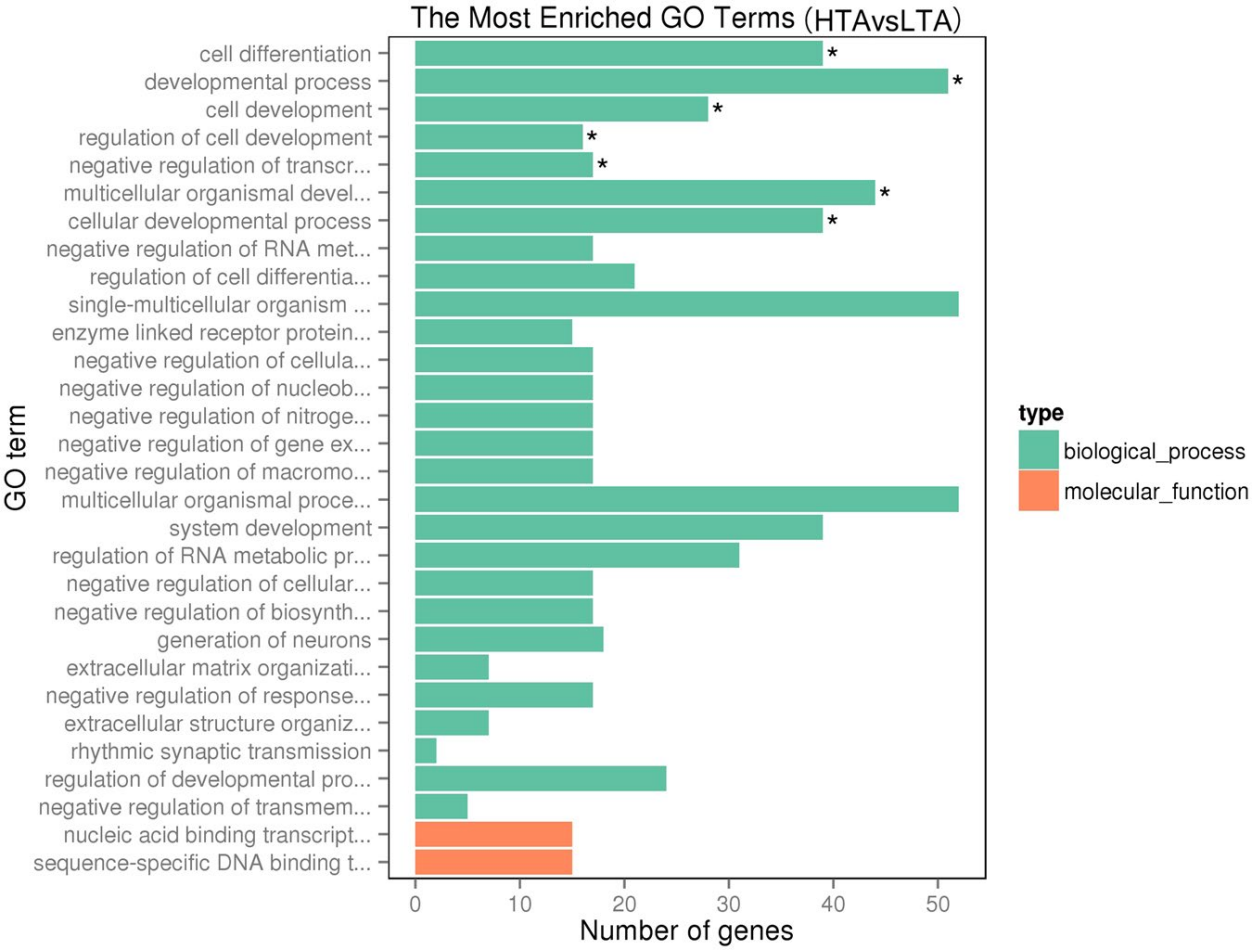
Output of Gene Ontology (GO) analysis. *P < 0.05.

To define the biological functions of the 220 DEGs, GO term and KEGG pathway analyses were carried out. GO enrichment analysis revealed large lists of enriched genes corresponding to significant GO terms in the categories of biological process, cellular component and molecular function. The top 10 most significantly enriched GO terms in the high versus low albumen groups are listed in Supplementary Table S3 and included developmental process, cellular developmental process and differentiation, multicellular organismal development and process, single-multicellular organism process, regulation of cell development, system development, and extracellular matrix organisation. The GO analysis revealed that up-regulated genes in HTA were involved in a wide range of regulatory functions (Fig. 3).

Moreover, the top 10 enriched KEGGs and DEGs were belong to the following pathways: glycosphingolipid biosynthesis, focal adhesion, extracellular matrix (ECM)-receptor interaction, cytokine-cytokine receptor interaction, regulation of actin cytoskeleton, neuroactive ligand-receptor interaction, cell adhesion molecules, synthesis and degradation of ketone bodies, cardiac muscle contraction, and folate biosynthesis (Supplementary Table S4).

### Candidate genes

Based on the combination of the significance levels and expression levels of the DEGs, the GO and pathway results suggest *ST3GAL4*, *FUT4*, *ITGA2*, *SDC3*, *PRLR*, *CDH4* and *GALNT9* as promising candidate genes for thick albumen synthesis, transport and metabolism during the process of egg laying. The details of the above candidate genes identified in the comparison of the HTA and LTA groups are listed in Table 2. To investigate the interactions between the candidate genes, we generated an interaction network based on the STRING 10 database. The genes that were up-regulated in the pathway network (Supplementary Fig. S1) were *ST3GAL4*, *SDC3*, and *CDH4*, while the down-regulated genes were *FUT4*, *ITGA2*, *PRLR*, and *GALNT9*.

**Table 2:** Summary of candidate genes involved in albumen synthesis, based on differential expression in magnum tissue samples between the HTA group and LTA group.

Symbol	CHR	Ensembl gene ID	Log2 fold change	Gene name	P-value
*ST3GAL4*	24	ENSGALG00000001100	1.09	Galactoside 2, 3 sialyltransferase 4	2.76E–02
*FUT4*	1	ENSGALG00000022718	−1.55	Fucosyltransferases 4	3.23E–02
*ITGA2*	Z	ENSGALG00000014903	−1.64	Integrin 2	1.86E–02
*SDC3*	23	ENSGALG00000000569	1.03	Syndecan 3	1.55E–02
*PRLR*	Z	ENSGALG00000003446	−3.54	Prolactin receptor	8.14E–06
*CDH4*	20	ENSGALG00000005102	1.32	Cadherin 4	4.49E–03
*GALNT9*	15	ENSGALG00000002242	−1.19	N-acetylgalactosaminyltransferase 9	2.50E–02

### Quantitative RT-PCR validation

**Figure 4:**
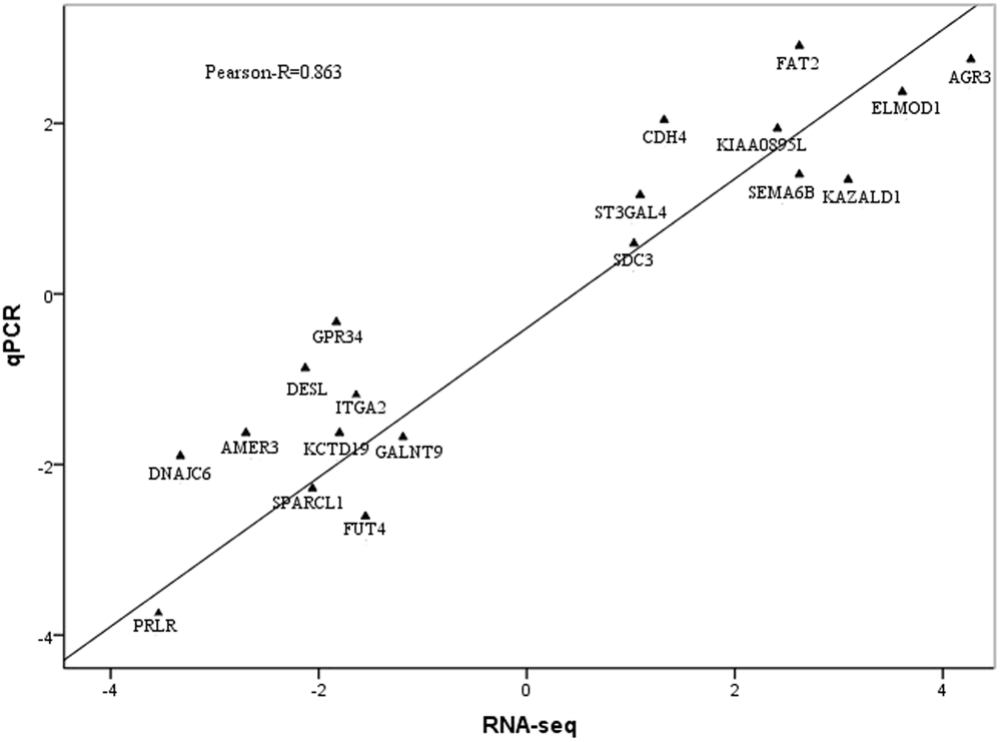
Correlations of the mRNA expression levels of 19 random differentially expressed genes in magnum tissues between the high- and low-thick egg albumen groups based on RNA-Seq and qRT-PCR analyses. The *x*- and *y*-axes show the log2 (ratio of mRNA levels) values measured via RNA-Seq and qRT-PCR, respectively. The DEGs indicated with dots were identified between the high- and low-thick albumen groups (HTA vs. LTA).

To validate the RNA-Seq results, 19 random DEGs, including nine up-regulated genes (*AGR3, ELMOD1, KAZALD1, SEMA6B, FAT2*, *KIAA0895L, ST3GAL4, SDC3* and *CDH4*) and ten down-regulated genes (*KCTD19, PRLR, DNAJC6, AMER3, DSEL, SPARCL1*, *GPR34, FUT4, ITGA2* and *GALNT9*) were selected for quantitative reverse transcription polymerase chain reaction (qRT-PCR) analysis. Comparisons of the expression levels of these 19 genes determined through qRT-PCR, normalised to glyceraldehyde 3-phosphate dehydrogenase (*GAPDH*), and RNA-Seq are shown in Supplementary Fig. S2. Relatively strong correlations were observed between the mRNA expression levels from qRT-PCR and RNA-Seq, with a Pearson correlation coefficient of 0.863 (Fig. 4). Therefore, the relative fold change of gene expression was consistent between the RNA-Seq and qRT-PCR data.

## Discussion

The magnum, as a part of the oviduct, is one of the most important reproductive structures and plays an important role in regulating the formation of egg albumen^3^. Egg albumen is mainly composed of numerous proteins in an aqueous solution (e.g., ovalbumin, ovomucin and globulin)^16^. The characteristic gel structure of thick egg albumen is considered to be attributable to the ovomucin protein fraction^6^. Furthermore, the proteins of the egg white or albumen of avian eggs are synthesised and stored in the mucosal cells of the magnum^3^. Therefore, knowledge of the magnum transcriptome might assist in the identification of genes that are important for the regulation of thick egg albumen in chickens.

In this study, the expression levels of 220 genes were found to differ significantly between the two groups (high versus low-thick white levels) according to the transcriptome profiles of magnum tissue samples. Among the top 10 most highly expressed genes (*AGR3*, *SEPT4*, *ELMOD1*, *FAM178B*, *KAZALD1*, *PRLR*, *DNAJC6*, *AMER3*, *DSEL* and *SPARCL1*) detected in the samples, most have been associated with various functions (e.g., tumor differentiation^17^, cytoskeletal alteration and cell apoptosis^18^, cell growth and differentiation^19^, epithelial–mesenchymal transition^20^ and Wnt signalling^21^). It is important to note that *PRLR* was significantly down-regulated in the high-thick white group (*P* < 0.01, log2 fold change = 3.54). The prolactin receptor plays an important role in the prolactin signal transduction cascade^22^ and is associated with many reproductive traits in poultry.

Based on our GO and KEGG analyses, we found that the molecular functions of most DEGs in the HTA and LTA individuals fell within the biological process category (cellular and organismal developmental processes). It has been widely accepted that the molecular regulation of animal phenotypes is highly complex, and the relationships between genes and phenotypes are “one-to-many” or “many-to-one”^23,24^. A total of 45 KEGG pathways were associated with the DEGs, four of which were overrepresented and related to protein metabolism, glycosphingolipid biosynthesis, focal adhesion, ECM-receptor interactions and cytokine-cytokine receptor interactions.

Glycosphingolipid biosynthesis plays crucial roles in many biological functions, and glycosphingolipids are reportedly synthesised at high levels in early chick embryos^25^. Focal adhesions serve as stable sites of tight adhesion, providing a structural link between the actin cytoskeleton and ECM components^26^. This pathway is likely involved in the structure and secretion of protein from thick albumen. The ECM consists of a complex mixture of structural and functional macromolecules and plays an important role in the maintenance of cell and tissue structure and function. Glycoproteins are a class of ECM molecules that includes interactive glycoproteins, which exist in several variant forms and possess multiple binding domains that are capable of binding collagen and proteoglycans^26^. The major components of egg albumen are various glycoproteins, such as ovalbumin, ovomucin and conalbumin^16^, and the thick white typically contains a higher proportion of ovomucin^27^. Therefore, it is reasonable to assume that ECM-receptor interactions are closely associated with the formation of thick albumen. The interaction of cytokines and cytokine receptors is an important pathway in the bidirectional network between the immune and the neuroendocrine systems that can modulate the responses of all endocrine axes^28^. Therefore, based on the results of RNA-Seq combined with the statistical significance and expression levels of DEGs and the pathway results, we identified *ST3GAL4*, *FUT4*, *ITGA2*, *SDC3*, *PRLR*, *CDH4* and *GALNT9* as novel promising candidate genes associated with thick albumen protein synthesis, transport and metabolism.

Galactoside 2,3 sialyltransferase 4 (*ST3GAL4*) is a member of the sialyltransferase family that plays important roles in many biological functions^29^. Glycosylated forms may enhance the hydrophilicity and stability of proteins. *ST3GAL4* appears to play a prominent role in the regulation of glycoproteins^30^, and glycoproteins have been recognised as an important component of egg albumen proteins. Fucosyltransferase IV (*FUT4*) is the key enzyme involved in the synthesis of the LeY oligosaccharide. LeY is a difucose oligosaccharide that is highly expressed in epithelial tissues, such as those of the breast, ovary and oviduct, and is responsible for the synthesis of glycoproteins^31^. Fucosylation is the final step in the glycosylation of proteins, which produces glycans that are involved in tumour glycoprotein development^32^. Glycoproteins, secreted from the epithelium of the oviduct in chickens, are important for the maturation and fertilisation capacity of egg albumen.

Integrin 2 (*ITGA2*) is a heterodimeric protein that mediates cell-ECM interactions. The ECM consists mainly of glycoproteins and collagen, and it provides primary structural support for tissues and cells during embryonic development^33^. The expression of *ITGA2* is associated with progesterone withdrawal^34^. The role of progesterone has been most extensively studied in female reproductive tissues (ovary^35^, oviduct^36^ and mammary gland^37^) of various species. In addition, progesterone induces the expression of egg white proteins, reduces myometrial contractility, and facilitates the processing of the eggs, formation of the shell, and deposition of egg white proteins^38^. The *ITGA2* gene functions through interaction with progesterone for the regulation of egg white protein.

Syndecan-3 (*SDC3*) is a member of the syndecan family of type I transmembrane proteins, which possess a large extracellular domain with a signal peptide and varying amounts of glycosaminoglycan (GAG) attachment sites^39,40^. It has recently been associated with growth traits in livestock^41^. The functions of syndecans (SDCs) are diverse, ranging from the involvement in cell adhesion to the organisation of cell matrix adhesion and signalling, and they provide interesting insights into protein biology^42,43^. Considering its effects on glycoproteins, *SDC3* appears to play a role in regulating the accumulation of proteins during the process of egg formation.

The prolactin receptor (*PRLR*) is an important regulatory gene located on the Z sex chromosome of chickens. The *PRLR* gene is expressed in many tissues, including the testes, ovaries, deferent ducts, oviduct and kidneys^44^, and it has been reported to be closely associated with many production traits in poultry^45^. In birds, *PRLR* inhibits gonadotropin release from the anterior pituitary, thereby inhibiting ovum development and ovulation, leading to ovarian regression^46^. In the present study, the *PRLR* gene was down-regulated in the HTA group, showing a log2 fold change of 3.54, suggesting that low expression of this gene might be associated with a high percentage of thick albumen in eggs.

The *CDH4* (R-cadherin) gene was first isolated from the chicken retina and is referred to as retinal cadherin^47^. Cadherins are a family of cell surface molecules that mediate cell-cell adhesion in a variety of tissues, and the functions of cadherins have been studied in different species. For example, in the nervous system, the proteins Cdh2 and Cdh4 are expressed along the neurites of the developing chicken brain^48^. Epithelial cadherin (E-cadherin) is present in relatively high concentrations in the epithelial cells of the bovine oviduct and in bovine gametes and participates in cell-cell interactions that are involved in fertilisation-related events^49^. In addition, *CDH4* has been reported to regulate the formation of epithelia^50^.

N-acetylgalactosaminyltransferase 9 (*GALNT9*) encodes a member of the polypeptide N-acetylgalactosaminy-ltransferase family of enzymes^51^ that participates in the biosynthesis of O polyose and has been found to play an important role in glycobiology^52^. In oviductal epithelial cells, proteins can be glycosylated, resulting in the formation of glycoproteins that are stored in the form of albumen in eggs. Thus, the *GALNT9* gene could influence the synthesis of thick albumen through the regulation of glycometabolism.

The construction of gene networks and pathways is an effective strategy for elucidating the mechanisms underlying the genetic variability of egg protein traits. Various genes generally cooperate with each other to carry out their biological functions^53^. Among the DEGs identified in the magnum of chickens with different percentages of thick egg albumen, the significant pathways and GO terms were mainly associated with the biosynthesis, transport and metabolism of proteins. The data obtained in the present study indicate that related genes participate in the regulation of oviduct-specific glycoproteins, and other oviduct-specific proteins exert effects on the formation of the egg white. Glycoproteins have been extensively identified in the glandular and epithelial cells of the oviduct^54^. Thus, glycoproteins are critical because of the importance of and need for egg proteins.

## Conclusions

To our knowledge, this is the first study to apply RNA-Seq technology to analyze the expression profiles of chicken magnum tissues with divergent thick egg albumen proportions. We identified 150 up-regulated genes and 70 down-regulated genes in the HTA group. Based on analysing the function of these genes, seven candidate genes (*ST3GAL4*, *FUT4*, *ITGA2*, *SDC3*, *PRLR*, *CDH4* and *GALNT9*) can be considered promising novel candidates for the synthesis and metabolism of thick egg albumen. The reproductive roles of these seven genes should be further investigated in specific magnum cells (such as glandular and epithelial cells) to determine their specific mechanisms and functions.

These novel insights into the metabolic transcriptome of chicken magnum tissues provide a sound basis for future investigations into the gene networks involved in the formation of proteins in thick egg albumen and facilitate the understanding of the molecular synthesis of egg albumen.

## Materials and Methods

### Ethics statement

All protocols and procedures involving animals were performed in accordance with the Regulations for the Administration of Affairs Concerning Experimental Animals (Ministry of Science and Technology, China) and were approved by the Committee for the Care and Use of Experimental Animals at Anhui Agricultural University (permit No. AHAU20101025). During the experimental period, the birds were reared in the same environment, were fed the same diet *ad libitum* and were humanely sacrificed.

### Animal husbandry and magnum collection

A pure line of Rhode Island white layers with complete pedigrees (G4 to G6), maintained in a breeding farm at Anhui Rongda Poultry Development Co., Ltd, China, were used in this study. The experimental chickens were selected as closed populations for six generations according to their appearance, growth and egg production within each generation. At 18 weeks of age, 404 hens were randomly selected and transferred to individual cages to record individual egg composition traits. All chickens were kept under a light/dark cycle of 16 h light and 8 h darkness (16 L: 8D) and were given free access to feed and water. The feeding procedure and the ingredient composition of the basal diet were furnished by the company (Supplementary Table S5). Eggs were collected over three to five successive days to measure relative egg composition traits at 30, 40 and 50 weeks of age. An average of three eggs per bird was used in the analyses. The procedures for measuring the egg composition and for the separation of thick and thin white were carried out as previously described^55,56^. All eggs were kept in the same storage room and measured within 12 h after egg laying.

At the age of 50 weeks, 9 females were slaughtered 1–3 h after laying, four from the HTA group and five from the LTA group (Supplementary Table S6); these groups consisted of chickens producing eggs with a high- or low-thick albumen level, respectively. The high- and low-thick albumen groups were judged by the ratio of thick to thin white (Supplementary Table S6). For RNA isolation, magnum tissues were rapidly harvested from the chickens’ carcasses, frozen in liquid nitrogen and stored at −80 °C until further processing.

### Total RNA extraction, cDNA library preparation and sequencing

A total of 9 cDNA libraries were sequenced from the magnums of the HTA and LTA groups. The RNA sequencing reads have been submitted to the National Center for Biotechnology Information (NCBI) Short Read Archive (SRA) database (Accession no. SRP108815, Bioproject: PRJNA389645).

The magnum samples of the selected birds were ground in a frozen state in liquid nitrogen. Total RNA was extracted from 70–100 mg of the samples using the TRIzol reagent (Tiangen Biotech Co., Beijing, China). The quality of the total RNA was checked using the Agilent 2100 Bioanalyzer System (Santa Clara, CA, USA). Ten cDNA libraries were constructed from the HTA and LTA groups, respectively, using the Illumina TruSeq stranded RNA sample preparation kit (Transgen Biotech Co., Beijing, China), according to the manufacturer’s protocol. The mRNA was extracted from total RNA using oligo (dT) magnetic beads and sheared into short fragments of approximately 200 bases. These fragmented mRNAs were then used as templates for cDNA synthesis. The cDNAs were subsequently amplified to complete library preparation using PCR, and the cDNA library was paired-end sequenced using the Illumina HiSeq^TM^ 2500 platform.

### Differential expression analysis

Clean reads were obtained by removing low-quality reads and/or trimming the adaptor sequences from raw reads. These processed reads were mapped to the *G. gallus* genome (http://ftp.ensembl.org/pub/release-83/fasta/gallus_gallus/dna/) using TopHat software^57^, allowing up to two base mismatches. The clean reads were employed for gene expression analysis. The mapped reads were used for further transcript annotation and for calculating expression levels using the fragments per kilobase per million reads (FPKM) method and the HTseq software package (version 0.6.1). The DEGs were identified with the DESeq software package (version 1.12.0)^58^. The Benjamini–Hochberg false discovery rate was employed to correct the *P*-values. Only genes with a *P*-value < 0.05 were considered differentially expressed. The k-means method was used to cluster the DEGs based on their relative expression levels.

### Bioinformatics analysis of DEGs

The DEG lists were submitted to the GO and KEGG databases for enrichment analysis. The GO terms and KEGG pathways (http://www.genome.jp/kegg/) showing *P*-values of less than 0.05 were considered significantly enriched among the DEGs. The STRING 10 database (http://string-db.org/) was employed to identify associations between the candidate genes identified in our study. Gene networks were constructed using Cytoscape (version 3.5.0).

### Confirmation of RNA-Seq results via qRT-PCR

To validate the RNA-Seq data, qRT-PCR was performed on 19 randomly selected DEGs. Total RNA was reverse-transcribed into cDNA using the PrimeScript RT reagent kit with gDNA Eraser (TaKaRa), according to the manufacturer’s instructions. Primers were designed according to sequence information from the NCBI database using Primer Premier 5.0 software (Applied Biosystems) (Supplementary Table S7). qRT-PCR was performed in a final reaction volume of 15 µL with the SYBR^®^ Green PCR Master Mix Kit (TaKaRa, Osaka, Japan) in the CFX96 Real-Time PCR Detection System (Bio-Rad, Hercules, CA, USA), using the following protocol: 95 °C for 10 min; 40 cycles of 95 °C for 15 s and 60 °C for 1 min. All reactions were performed in triplicate for each sample, and GAPDH was employed as a reference gene for the normalisation of gene expression levels. The relative gene expression values were calculated using the 2^−ΔΔCt^ method^59^. Comparisons of gene expression levels based on prolificacy levels were performed using the *t*-test, and the correlations between the qRT-PCR and RNA-Seq measurements were calculated.

## Electronic supplementary material

Supplementary Information

Supplementary Table
