# Supplementary material for: RNA-Seq reveals seven promising candidate genes affecting the proportion of thick egg albumen in layer-type chickens
Source: Sci Rep. 2017 Dec 22;7:18083. doi: 10.1038/s41598-017-18389-5 (PMC5741707; doi:10.1038/s41598-017-18389-5)
Supplement: Supplementary file 1 — Supplementary Information [file 41598_2017_18389_MOESM1_ESM.pdf]

RNA-Seq reveals seven promising candidate genes affecting the proportion of thick egg albumen in layer-type chickens

Yi Wan[1], Sihua Jin[1], Chendong Ma, Zhicheng Wang, Qi Fang, Runshen Jiang[*]

College of Animal Science and Technology, Anhui Agricultural University, Hefei 230036, China

[1]These authors contributed equally to the work.

[*]Corresponding author: Runshen Jiang, College of Animal Science and Technology, Anhui Agricultural University, No. 130 Changjiang West Road, Hefei 230036, China

E-mail: jiangrunshen@ahau.edu.cn

Supplementary Table S1. Basic statistics for RNA-Seq reads of the magnum samples from chickens with high- and low-thick egg albumen levels

Sample name[1]	Raw reads	Clean reads	Q20 (%)[2]	Q30 (%)[3]	GC content (%)[4]	Multiple mapped reads	Uniquely mapped reads	Mapping rate (%)[5]	Exons, %	Intron, %	Intergenic, %
H1	68554478	66438872	97.29	93.32	47.24	1122242 (1.69%)	55828747 (84.03%)	85.72	84.20	3.20	12.60
H2	61092766	60077470	97.60	93.93	46.60	753734 (1.25%)	48804032 (81.24%)	82.49	81.70	4.10	14.20
H3	52353036	51647840	97.88	94.52	46.24	584508 (1.13%)	44102522 (85.39%)	86.52	83.90	5.80	10.30
H4	47391428	46738774	97.95	94.72	46.12	446993 (0.96%)	39007512 (83.46%)	84.41	87.00	4.40	8.60
L1	53106586	51851054	98.04	94.81	45.05	476545 (0.92%)	44198226 (85.24%)	86.16	89.10	2.90	8.00
L2	56726408	55979582	97.93	94.65	46.44	671365 (1.2%)	46219049 (82.56%)	83.76	82.60	5.00	12.40
L3	48398688	47771016	98.03	94.85	46.11	660745 (1.38%)	40690537 (85.18%)	86.56	85.00	4.50	10.50
L4	51920788	50552704	97.93	94.61	46.02	945393 (1.87%)	42634304 (84.34%)	86.21	84.90	4.80	10.30
L5	56857800	55383926	97.79	94.35	47.07	720923 (1.3%)	46604597 (84.15%)	85.45	83.50	4.30	12.20

[1]H1, H2, H3, H4 and L1, L2, L3, L4, L5 are replicates of the HTA and LTA groups. HTA represents the extreme high-thick albumen group, and LTA represents the

extreme low-thick albumen group.

[2]Q20: the proportion of bases with phred base quality score greater than 20; i.e., the proportion of read bases whose error rate is less than 1%.

[3]Q30: the proportion of bases with phred base quality score greater than 30; i.e., the proportion of read bases whose error rate is less than 0.1%.

[4]GC content: guanine-cytosine content

[5]*Gallus gallus* is used as the reference genome annotation to classify the mapping tags into different regions. The ratio was calculated as the number of tags in each region divided by the total tags in the whole genome.

Supplementary Table S3. Top 10 most significantly enriched Gene Ontology (GO) terms for differentially expressed genes (DEGs) in the magnum of chickens

GO term	Description	P-value	Up DEGs	Down DEGs	DEGs
GO:0032502	Developmental process	3.44E-05	51	17	68
GO:0048869	Cellular developmental process	5.10E-05	39	13	52
GO:0007275	Multicellular organismal development	5.25E-05	44	15	59
GO:0044707	Single-multicellular organismal process	7.35E-05	52	21	73
GO:0030154	Cell differentiation	7.53E-05	39	9	48
GO:0032501	Multicellular organismal process	1.09E-04	52	22	74
GO:0048468	Cell development	4.96E-04	28	4	32
GO:0060284	Regulation of cell development	5.69E-04	16	1	17
GO:0048731	System development	5.87E-04	39	13	52
GO:0030198	Extracellular matrix organisation	5.89E-04	7	1	8

Supplementary Table S4. Top 10 significantly enriched Kyoto Encyclopedia of Genes and Genomes pathways for differentially expressed genes (DEGs)

Pathway ID	Pathways	P-value	DEGs	Background number
gga00601	Glycosphingolipid biosynthesis	0.0146	2	21
gga04510	Focal adhesion	0.0181	5	184
gga04512	ECM-receptor interaction	0.0233	3	73
gga04060	Cytokine-cytokine receptor interaction	0.0474	4	165
gga04810	Regulation of actin cytoskeleton	0.0583	4	177
gga04080	Neuroactive ligand-receptor interaction	0.0634	5	261
gga04514	Cell adhesion molecules (CAMs)	0.0657	3	112
gga00072	Synthesis and degradation of ketone bodies	0.0779	1	9
gga04260	Cardiac muscle contraction	0.0892	2	60
gga00790	Folate biosynthesis	0.1000	1	12

Supplementary Table S5. Ingredient composition and nutrient levels of the basal diet

Ingredient	Content (%)	Nutrient levels (calculated values)	
Corn	55.92	Metabolisable energy (kcal/kg)	3073
Soybean oil	4.55	Crude protein (%)	19.22
Soybean meal	29.77	Crude fibre (%)	3.15
Salt	0.30	Phosphorus (%)	0.52
$_{D,L}$-Methionine	0.20	Calcium (%)	0.96
Limestone	6.0	Lysine (%)	1.05
Dicalcium phosphate	1.86	Methionine (%)	0.52
Calcium carbonate	1.10	Methionine + cysteine (%)	0.76
Premix[1]	0.30		
Total	100.00		

[1]Premix provided per kg of diet: Cu, 10 mg; Fe, 50 mg; Mn, 60 mg; Zn, 65 mg; Se, 0.40 mg; vitamin A, 9000 IU; vitamin D_3, 3100 IU; vitamin E, 25 IU; vitamin B_1,

2.5 mg; vitamin B_2, 5 mg; vitamin B_{12}, 0.02 mg; biotin, 0.1 mg; folacin, 1 mg; pantothenic acid, 12 mg; nicotinic acid, 38 mg; and pyridoxine 3.5 mg.

Supplementary Table S6. Chicken groups with high and low proportions of thick albumen

Group	Sample	Traits							
		Egg weight	Shell strength	Yolk (%)	Thick white (%)	Thin white (%)	Thick:Thin	Albumen height	Haugh units
HTA, n=5	HTA4	65.57±2.90	4.24±0.51	28.73±2.97	44.65±4.30	23.15±2.47	1.96±0.33	8.81±1.25	91.88±7.21
	HTA1	62.31±3.96	3.29±0.86	24.93±2.79	45.20±4.29	25.45±4.65	1.85±0.47	9.65±1.35	96.66±5.63
	HTA2	54.75±2.31	3.60±0.79	27.65±3.26	41.19±5.35	23.39±2.84	1.79±0.34	7.14±1.48	84.94±7.85
	HTA3	53.60±2.60	3.20±0.96	31.40±2.34	40.00±4.63	23.76±4.96	1.77±0.50	7.43±0.76	87.81±4.04
**Overall mean**	**HTA**	**59.26±5.90**	**3.56±0.88**	**28.21±3.65**	**42.85±4.94^{A}**	**24.01±3.94^{A}**	**1.84±0.41^{A}**	**8.38±1.53**	**90.97±7.22**
LTA, n=5	LTA4	60.59±4.08	3.73±0.90	26.40±3.48	32.76±4.55	35.06±3.47	0.94±0.16	9.36±1.02	96.06±4.62
	LTA1	56.42±1.83	3.38±0.61	27.03±2.55	30.90±4.24	36.64±4.41	0.86±0.16	8.01±0.87	90.23±4.51
	LTA3	60.76±0.80	3.39±0.27	29.96±2.47	28.66±5.02	34.06±2.65	0.85±0.17	7.97±0.54	88.96±3.24

	LTA2	57.87±2.92	2.91±1.90	29.12±2.24	29.93±5.27	36.36±2.78	0.83±0.20	7.80±0.71	88.75±3.71
	LTA5	53.01±1.85	3.74±0.89	28.72±2.61	28.79±5.30	37.06±5.67	0.81±0.22	8.08±1.27	91.30±6.53
**Overall mean**	**LTA**	**57.44±3.79**	**3.42±1.10**	**28.17±2.87**	**30.26±4.88^{B}**	**35.98±4.00^{B}**	**0.86±0.18^{B}**	**8.25±1.07**	**91.15±5.27**

Values represent means±s.e.m. HTA represents the extreme high-thick albumen group, and LTA represents the extreme low-thick albumen group. Means in the same column with different letters (A and B) differ at P <0.01.

Supplementary Table S7. PCR primers for qRT-PCR validation of 19 differentially expressed genes in magnum samples from the high- and low-thick egg albumen groups

Gene name	Log_2 fold change by RNA-Seq	Forward primer sequence	Reverse primer sequence	Amplicon (bp)	Tm (°C)
AGR3	4.27	TACGAACCACAAGACATACCA	GCAGAGAAAGAGGCTAAATAAAA	165	56
ELMOD1	3.61	GAAGGCGATGTGATTTGATT	CGATTTGCCATTTCTTGTTT	198	54
KAZALD1	3.09	TGCTCTCCTTCCCTTCTTTC	CCCCTTTTATTTGCTCGCT	103	56
SEMA6B	2.62	GGATGAAGGGGAAACACGA	GCACAGATGGGGTTGAAGG	112	58
FAT2	2.62	GCTTTGCCTCCTTTGTCTT	CTCTCCATCTTTCCCCTTATC	107	55
KIAA0895L	2.41	ACCTCTGCTCTCCCTGCTC	CCTACCCCGAGTCCTTTGT	127	60
ST3GAL4	1.09	TGAAGCAGCCAAGGAAGGT	AGGTCGCAGAAGTGGAGAG	79	59
SDC3	1.03	CATCAGCAACAGGACACAGT	GGAGCAGGAGCCAAGAAG	193	58
CDH4	1.32	CTGCTCCACCACCACTCT	TGCTGCTCGCTTACCTTCTA	79	58

KCTD19	-1.80	ATGTGGGGCTGAAGAAAAG	TTGGGAGAGTAGTTTTGATGG	110	55
PRLR	-3.54	TTTTGGTGGAAGATGAAGAAG	CCTGTTTTGTTTGACCTGTG	140	55
DNAJC6	-3.33	ATCCAACCATTCTCTTCTCCT	CACTCTGTCCAACATTCCTG	111	56
AMER3	-2.70	GCTCTGAAAACCCTTTGCT	TCACTTCTGTCCTCCCTCTC	109	57
DSEL	-2.13	TGGGAAAATGGTAAAGGAAA	AATGGGACAACACAGAAACA	115	54
SPARCL1	-2.06	CACAAGAAGCACAGCAACA	TTCACCCCATTCCTCATCT	186	56
GPR34	-1.83	AGAAGAACGCAATGACAGAAG	AGGAAAGTATCAAAAGCAGGAA	84	56
FUT4	-1.55	CGCCGACTCCTTCATCCA	CAGTAATGCTCATCCCAGAAAGAC	159	59
ITGA2	-1.64	ATTGGTGTTGTGATTGGCAGTAT	CAGTCTTTGTCTTTCGTGAGTTCT	161	59
GALNT9	-1.19	TTACACAGAACGGTCCAATCATC	CTCAGCCTCAGCAGTCAATG	169	58
GAPDH		GGAGAAACCAGCCAAGTATG	ATCAAAGGTGGAGGAATGG	142	55

Supplementary Figure S1. Relationships between the seven candidate genes

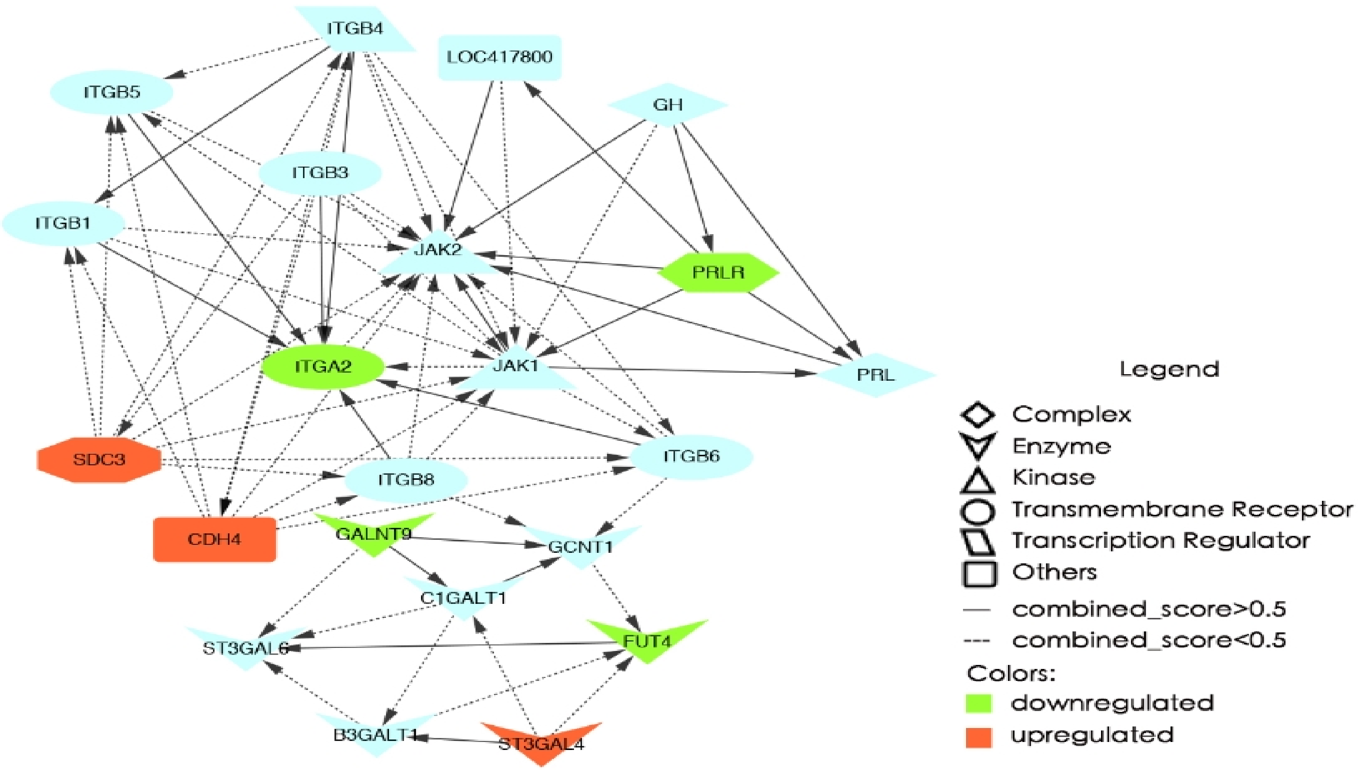

Dotted lines represent indirect interactions, and solid lines represent direct interactions. The genes highlighted in red were up-regulated, while those highlighted in green were down-regulated in the HTA group. The genes highlighted in blue were DEGs but were not candidate genes that were predicted to be involved in the

network.

Supplementary Figure S2. Comparisons between qRT-PCR and RNA-Seq measurements of the expression abundance of 19 random differentially expressed genes

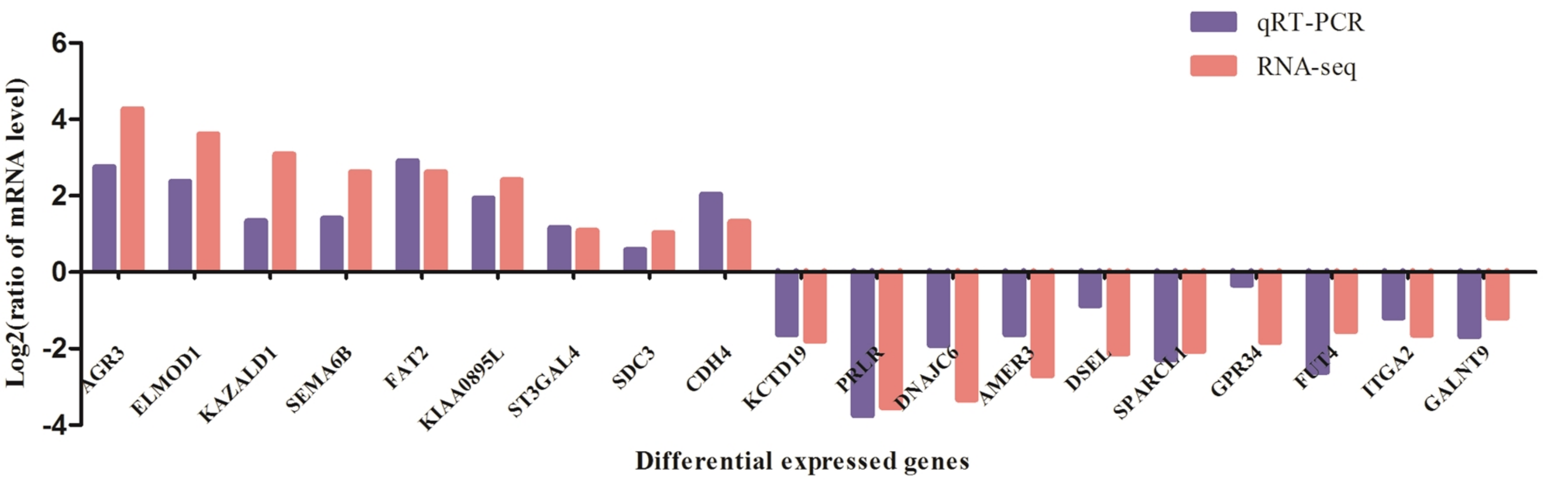

The log2 (ratio of mRNA level) value is the logarithm of the normalised mRNA expression level ($2^{-\Delta\Delta Ct}$) in HTA relative to the normalised mRNA expression level ($2^{-\Delta\Delta Ct}$) in LTA. A log2-ratio > 0 indicates up-regulation in HTA compared with LTA; a log2-ratio < 0 indicates down-regulation in HTA compared with LTA.
