# Supplementary material for: RNA-Seq reveals seven promising candidate genes affecting the proportion of thick egg albumen in layer-type chickens
Source: Sci Rep. 2017 Dec 22;7:18083. doi: 10.1038/s41598-017-18389-5 (PMC5741707; doi:10.1038/s41598-017-18389-5)
Supplement: Supplementary file 2 — Supplementary Table [file 41598_2017_18389_MOESM2_ESM.pdf]

RNA-Seq reveals seven promising candidate genes affecting the proportion of thick egg albumen in layer-type chickens

Yi Wan[1], Sihua Jin[1], Chendong Ma, Zhicheng Wang, Qi Fang, Runshen Jiang*

College of Animal Science and Technology, Anhui Agricultural University, Hefei 230036, China

[1]These authors contributed equally to the work.

*Corresponding author: Runshen Jiang, College of Animal Science and Technology, Anhui Agricultural University, No. 130 Changjiang West Road, Hefei 230036, China

E-mail: jiangrunshen@ahau.edu.cn

Supplementary Table S2. List of differentially expressed genes in magnum tissues between high and low thick egg albumen

Gene_id	readcount_HTA	readcount_LTA	log2FoldChange	pvalue	symbol	interpro description
ENSGALG00000015117	0	2.314826035	#NAME?	0.017914	DRD3	Dopamine receptor family||G protein-coupled receptor, rhodopsin-like||GPCR, rhodopsin-like, 7TM||7TM GPCR, olfactory receptor/chemoreceptor Srsx||Dopamine D3 receptor
ENSGALG00000026628	2.92012785	0	Inf	0.0076405	-	PMP-22/EMP/MP20/Claudin superfamily||Voltage-dependent calcium channel, gamma-5 subunit||Voltage-dependent calcium channel, gamma subunit||Voltage-dependent calcium channel, gamma-7 subunit
Novel01223	52.48594314	0	Inf	0.00046784	--	PREDICTED: SH3 domain-containing protein C23A1.17-like isoform X2 [Gallus gallus]
ENSGALG00000019651	3.531367872	0.183542252	4.266	0.0096015	AGR3	Thioredoxin-like fold||Domain of unknown function DUF255
ENSGALG00000028193	4.10807989	0.218821956	4.2306	0.004128	SEPT4	Ribosome biogenesis GTPase RsgA, putative||Septin-type guanine nucleotide-binding (G) domain||P-loop containing nucleoside triphosphate hydrolase
ENSGALG00000026617	3.059816406	0.170652531	4.1643	0.030071	-//-	
ENSGALG00000026620	2.966274089	0.218821956	3.7608	0.038813	-	Fibrinogen, alpha/beta/gamma chain, C-terminal globular domain||SRCR-like domain||SRCR domain
ENSGALG00000017169	20.39928163	1.672842365	3.6081	0.0045721	ELMOD1	Engulfment/cell motility, ELMO
ENSGALG00000028532	6.20182406	0.52692407	3.557	0.0089927	FAM178B	-
Novel01009	20.48751273	1.797402227	3.5108	0.00043585	--	PREDICTED: activator of basal transcription 1-like [Astyanax mexicanus]
ENSGALG00000013751	3.531699814	0.356271539	3.3093	0.027694	-//-	
Novel00417	3.279235679	0.361678022	3.1806	0.030792	--	PREDICTED: gap junction beta-5 protein isoform X1 [Gallus gallus]>gi|513221293|ref|XP_004947840.1| PREDICTED: gap junction beta-5 protein isoform X2 [Gallus gallus]
ENSGALG00000007837	12.66410638	1.487338541	3.0899	0.030904	KAZALD1	Insulin-like growth factor-binding protein, IGFBP||Immunoglobulin-like domain||Immunoglobulin subtype||Immunoglobulin||Immunoglobulin I-set||Immunoglobulin subtype 2||Insulin-like growth factor binding protein-related protein (IGFBP-rP), MAC25||Kazal domain
Novel01106	6.213907482	0.947584482	2.7132	0.009944	-//-	
ENSGALG00000001171	6.082962979	0.990597055	2.6184	0.0088266	SEMA6B	Plexin||Plexin-like fold||Sema domain
ENSGALG00000004320	15.75680351	2.569367224	2.6165	0.002217	FAT2	EGF-like calcium-binding domain||Epidermal growth factor-like domain||Cadherin-like||Concanavalin A-like lectin/glucanase domain||Cadherin||Laminin G domain
ENSGALG00000005941	9.125871874	1.689825866	2.4331	0.0037713	-	Immunoglobulin V-set domain||Immunoglobulin V-set, subgroup||Immunoglobulin subtype||Immunoglobulin-like domain
ENSGALG00000001779	9.067492036	1.711876342	2.4051	0.0049042	KIAA0895L	Protein of unknown function DUF1704
ENSGALG00000015395	24.90429249	4.805756211	2.3736	2.12E-05	-	Immunoglobulin subtype
ENSGALG00000025945	8.579482248	1.697443551	2.3375	0.023923	-	Avidin||Avidin/streptavidin
ENSGALG00000028258	5.162791084	1.125854698	2.1971	0.017007	-	von Willebrand factor, type C||Glycoprotein hormone subunit beta, cystine knot||IGFBP-related, CNN||Insulin-like growth factor-binding protein, IGFBP||Thrombospondin, type 1 repeat||Cystine knot, C-terminal
Novel00159	12.74000964	3.036160774	2.069	0.02836	-//-	
ENSGALG00000026476	18.51970745	4.822634853	1.9412	0.020118	TCF7L1	CTNNB1 binding, N-teminal||High mobility group box domain
ENSGALG00000010470	17.07696507	4.619144187	1.8864	0.034058	TCERG1L	FF domain||WW domain
Novel00724	4.709926029	1.31349073	1.8423	0.046194	-//-	
ENSGALG00000026499	38.58316842	11.21665079	1.7823	0.00019122	TNFRSF25	TNFR/NGFR cysteine-rich region||Death-like domain||Death domain
ENSGALG00000012285	39.71334719	11.55501431	1.7811	0.0001103	BAIAP2L2	SH3 domain||Variant SH3 domain||IMD/I-BAR domain
ENSGALG00000004386	296.3396873	86.39301142	1.7783	0.039536	Sox9	High mobility group box domain||Sox developmental protein N-terminal
ENSGALG00000026389	26.77081322	7.864831715	1.7672	0.0014517	-	Protein kinase domain||Protein kinase-like domain||Serine-threonine/tyrosine-protein kinase catalytic domain||Tyrosine-protein kinase, catalytic domain||Serine/threonine/dual specificity protein kinase, catalytic domain
ENSGALG00000029002	8.459095255	2.494214776	1.7619	0.033139	-	CD99 antigen-like protein 2
ENSGALG00000005903	66.11456071	19.74443149	1.7435	0.027615	NRIP3	Aspartic peptidase, DDI1-type||Aspartic peptidase domain
ENSGALG00000026501	31.87479189	9.521648812	1.7431	0.013774	-//-	
ENSGALG00000008263	10.74841946	3.226821451	1.7359	0.04743	FAR-1	Immunoglobulin-like domain||Immunoglobulin subtype||Immunoglobulin||Immunoglobulin V-set domain||Immunoglobulin subtype 2||Fibronectin type III||Immunoglobulin I-set
ENSGALG00000027907	11.83800591	3.564871501	1.7315	0.013247	NR2F1	Transcription factor COUP||Nuclear hormone receptor, ligand-binding||Zinc finger, nuclear hormone receptor-type||Steroid hormone receptor||Retinoid X receptor/HNF4||Nuclear hormone receptor, ligand-binding, core
ENSGALG00000009867	22.38875288	6.772122572	1.7251	0.00082787	WIF1	Wnt inhibitory factor (WIF)-1||EGF-like domain, extracellular||Epidermal growth factor-like domain||WIF domain
ENSGALG00000003345	9.03989901	2.765474852	1.7088	0.025899	-	Phospholipase C, phosphatidylinositol-specific, Y domain||Pleckstrin homology domain||PLC-like phosphodiesterase, TIM beta/alpha-barrel domain||Phospholipase C, phosphoinositol-specific, EF-hand-like||Phosphoinositide phospholipase C family||C2 domain||Phospholipase C, phosphatidylinositol-specific , X domain
ENSGALG00000007786	7.321116858	2.245859105	1.7048	0.037028	DOK5	Insulin receptor substrate-1, PTB||Pleckstrin homology domain
Novel00505	15.70214642	4.817422549	1.7046	0.020743	-//-	
ENSGALG00000015497	16.28537819	5.280786171	1.6248	0.0053756	VGLL3	TDU repeat||Vestigial/tondu
ENSGALG00000009724	8.461346071	2.769528163	1.6112	0.02285	GREM1	DAN||Gremlin precursor||Cystine knot, C-terminal
ENSGALG00000011369	10.71440732	3.533860492	1.6002	0.035929	LHX8	Zinc finger, LIM-type||Homeobox domain||Homeodomain-like
ENSGALG00000012495	14.38747238	4.791199298	1.5864	0.02035	-	SH3 domain||Variant SH3 domain||Nebulin||Nebulin repeat
ENSGALG00000009100	80.60070352	27.06883393	1.5742	0.044697	FSHR	GPCR, rhodopsin-like, 7TM||G protein-coupled receptor, rhodopsin-like||Leucine-rich repeat||Lutropin-choriogonadotropic hormone receptor||Follicle stimulating hormone receptor||7TM GPCR, serpentine receptor class v (Srv)||Glycoprotein hormone receptor family||Gonadotropin hormone receptor, transmembrane domain
ENSGALG00000026034	13.62745087	4.643965397	1.5531	0.032256	RGS17	RGS domain
ENSGALG00000026585	8.778854196	2.999338	1.5494	0.039325	-	Chemokine-like protein, FAM19A2
ENSGALG00000028031	9.470487194	3.2770079	1.5311	0.044603	C1QL1	Complement C1q protein||Tumour necrosis factor-like domain||Collagen triple helix repeat
ENSGALG00000003025	18.5884439	6.68336288	1.4758	0.021193	-	Major facilitator superfamily domain||Reduced folate carrier
ENSGALG00000000516	77.61541071	28.26771452	1.4572	0.03908	FANCA	Fanconi anaemia group A protein
ENSGALG00000024758	10.27272992	3.80098632	1.4344	0.043598	-//-	
ENSGALG00000016648	14.69768865	5.438973527	1.4342	0.023909	-	Type I phosphodiesterase/nucleotide pyrophosphatase/phosphate transferase||Sulfatase||Alkaline-phosphatase-like, core domain
Novel00444	13.17430884	4.911624911	1.4235	0.025093	--	hypothetical protein N335_04952, partial [Phaethon lepturus]
ENSGALG00000021074	19.73967114	7.382386631	1.4189	0.014935	MYOC	Olfactomedin-like
ENSGALG00000027089	21.01755892	8.01297908	1.3912	0.01939	ANKLE1	Ankyrin repeat-containing domain||Ankyrin repeat
ENSGALG00000003324	31.64214238	12.11039458	1.3856	0.012228	PRRX1	Homeodomain-like||Homeobox domain||OAR domain
ENSGALG00000012735	16.98174408	6.539877208	1.3766	0.024144	EDN1	Endothelin-like toxin||Bibrotoxin/Sarafotoxin-D
Novel01029	23.15859533	8.986492433	1.3657	0.027589	--	hypothetical protein N301_08835, partial [Charadrius vociferus]
ENSGALG00000000080	35.32744266	13.81743431	1.3543	0.040516	FER1L5	C2 domain
ENSGALG00000015192	9.917953526	3.910389807	1.3427	0.039164	GZMM	Peptidase S1A, chymotrypsin-type||Trypsin-like cysteine/serine peptidase domain||Peptidase S1
ENSGALG00000001734	13.8121771	5.488795162	1.3314	0.033757	-//-	
ENSGALG00000005102	25.86467818	10.3697828	1.3186	0.0044919	CDH4	Cadherin||Cadherin prodomain||Cadherin-like||Cadherin, cytoplasmic domain
ENSGALG00000023725	123.1723617	49.73098403	1.3085	0.021861	IER5	Immediate early response
Novel00040	374.5986525	154.1693837	1.2808	0.030993	--	PREDICTED: envelope glycoprotein gp95-like [Gallus gallus]
ENSGALG00000021087	87.80460477	36.26655098	1.2757	0.046133	-	Carboxylesterase, type B||Alpha/Beta hydrolase fold||Alpha/beta hydrolase fold-3
ENSGALG00000007269	57.37964784	23.70591033	1.2753	0.03413	GABRA3	Gamma-aminobutyric-acid A receptor, gamma subunit||Gamma-aminobutyric acid A receptor/Glycine receptor alpha||Gamma-aminobutyric-acid A receptor, alpha subunit||Neurotransmitter-gated ion-channel transmembrane domain||Neurotransmitter-gated ion-channel ligand-binding domain||Neurotransmitter-gated ion-channel
ENSGALG00000028386	41.96665448	17.5392641	1.2587	0.013224	SPEG	Fibronectin type III||Immunoglobulin I-set||Immunoglobulin subtype 2||Protein kinase-like domain||Tyrosine-protein kinase, catalytic domain||Serine-threonine/tyrosine-protein kinase catalytic domain||Immunoglobulin||Immunoglobulin subtype||Serine/threonine/dual specificity protein kinase, catalytic domain||Immunoglobulin-like domain||Protein kinase domain
ENSGALG00000020482	12.00687008	5.025196892	1.2566	0.046022	LPXN	Zinc finger, LIM-type
ENSGALG00000000616	1516.329565	635.62179	1.2543	0.025749	ELF3	Ets domain||Pointed domain||Sterile alpha motif/pointed domain
ENSGALG00000009965	17.8246812	7.563228762	1.2368	0.044087	MPL	Fibronectin type III||Growth hormone/erythropoietin receptor, ligand binding
ENSGALG00000004357	2144.351675	922.6955173	1.2166	0.033668	ANXA6	Annexin||Annexin, type VI||Annexin repeat||Annexin, type IV
ENSGALG00000023577	64.13825127	27.60743574	1.2161	0.0095826	TBX2	Transcription factor, T-box, region of unknown function||Transcription factor, T-box||Transcription factor, Brachyury||p53-like transcription factor, DNA-binding
Novel00471	81.88500684	35.43155149	1.2086	0.043538	--	PREDICTED: membrane primary amine oxidase isoformX2 [Gallus gallus]
ENSGALG00000024047	82.0470679	35.65619733	1.2023	0.0044373	MYCL	Myc-type, basic helix-loop-helix (bHLH) domain||Transcription regulator Myc, N-terminal||Transcription regulator Myc
ENSGALG00000029009	22.27876462	9.710501627	1.1981	0.033359	HMGA1	-
Novel00180	62.62051581	27.74528149	1.1744	0.011308	-//-	
ENSGALG00000012545	16.39698726	7.275179364	1.1724	0.039063	CYTIP	PDZ domain
Novel00818	53.52841617	24.03518102	1.1552	0.010601	--	PREDICTED: MHC class II regulatory factor RFX1 isoform X1 [Ictidomys tridecemlineatus]>gi|532101359|ref|XP_005336299.1| PREDICTED: MHC class II regulatory factor RFX1 isoform X2 [Ictidomys tridecemlineatus]
ENSGALG00000021634	63.23892545	28.59281425	1.1452	0.018982	MAP7D1	Microtubule-associated protein 7
Novel00585	41.28975578	18.67886383	1.1444	0.025466	--	PREDICTED: uncharacterized protein LOC102111320 [Pseudopodoces humilis]
ENSGALG00000023621	92.62435355	41.92563827	1.1436	0.018201	SFXN3	Tricarboxylate/iron carrier
Novel00690	157.6103486	71.81815251	1.1339	0.041766	--	PREDICTED: translation initiation factor IF-2-like [Gallus gallus]
ENSGALG00000025898	129.1656101	59.67265099	1.1141	0.044897	SMAD6	MAD homology, MH1||SMAD domain, Dwarfin-type||SMAD/FHA domain||MAD homology 1, Dwarfin-type
ENSGALG00000006584	101.8154247	47.49719998	1.1	0.032036	-//-	
ENSGALG00000001100	51.19778659	23.98709858	1.0938	0.027606	ST3GAL4	Sialyltransferase||Glycosyl transferase, family 29
ENSGALG00000029021	35.48301256	16.65134046	1.0915	0.021152	CACNA2D2	Voltage-dependent calcium channel, alpha-2/delta subunit, conserved region||VWA N-terminal||von Willebrand factor, type A||Cache domain
ENSGALG00000010121	24.36209085	11.52321588	1.0801	0.049132	-//-	
Novel00097	25.31816191	11.97777268	1.0798	0.031409	--	PREDICTED: class E basic helix-loop-helix protein 41, partial [Gallus gallus]
ENSGALG00000000521	39.21433658	18.5841025	1.0773	0.028072	SPIRE2	Zinc finger, FYVE/PHD-type||KIND
ENSGALG00000014179	67.12922823	31.92610762	1.0722	0.010014	SBK1	Serine/threonine-protein kinase, Sbk1||Protein kinase-like domain||Protein kinase domain||Tyrosine-protein kinase, catalytic domain||Serine-threonine/tyrosine-protein kinase catalytic domain||Serine/threonine/dual specificity protein kinase, catalytic domain
ENSGALG00000010145	168.2440198	80.12724133	1.0702	0.047085	MDGA1	Immunoglobulin V-set domain||Concanavalin A-like lectin/glucanase domain||Immunoglobulin||Immunoglobulin I-set||Fibronectin type III||Immunoglobulin subtype 2||Immunoglobulin-like domain||Immunoglobulin subtype||MAM domain
ENSGALG00000010996	573.8591168	273.5416785	1.0689	0.014899	IRF2BP2	Interferon regulatory factor 2-binding protein 1 & 2, zinc finger
ENSGALG00000029123	81.03755495	38.73109982	1.0651	0.020142	Rbfox3	RNA recognition motif domain||RNA binding protein Fox-1||Fox-1 C-terminal domain
ENSGALG00000010953	43.44469872	20.87136085	1.0577	0.018862	KANK4	Ankyrin repeat||Kank N-terminal motif||Ankyrin repeat-containing domain
ENSGALG00000027352	730.7063445	351.1425108	1.0572	0.0068785	-//-	
ENSGALG00000013254	117.7053424	56.76232715	1.0522	0.020886	LOXL3	Lysyl oxidase||SRCR-like domain||SRCR domain
ENSGALG00000023684	274.3411414	132.3486187	1.0516	0.039999	R3HCC1L	-
ENSGALG00000028294	69.69099743	33.75389746	1.0459	0.036097	VWA5B2	von Willebrand factor, type A
ENSGALG00000016774	45.77056313	22.18173492	1.045	0.016772	NPAS2	Myc-type, basic helix-loop-helix (bHLH) domain||PAS domain||PAS fold||PAS fold-3||Nuclear translocator||PAC motif
Novel00996	30.61094368	14.85506764	1.0431	0.036275	--	PREDICTED: LOW QUALITY PROTEIN: A-kinase anchor protein 8 [Meleagris gallopavo]
Novel01065	39.02206298	18.95683277	1.0416	0.036735	--	Carboxy-terminal kinesin 1 [Gallus gallus]
ENSGALG00000002081	18.25244001	8.883942758	1.0388	0.047122	MMP28	Peptidase M10A||Peptidase, metallopeptidase||Hemopexin-like domain||Peptidase M10A, stromelysin-type||Peptidase M10, metallopeptidase||Peptidoglycan binding-like||Hemopexin-like repeats
ENSGALG00000026081	510.3089805	248.9606763	1.0355	0.024469	RBM38	-
ENSGALG00000006609	272.8069631	133.3250398	1.0329	0.0036447	EPHB1	Ephrin receptor type-A /type-B||Ephrin receptor ligand binding domain||Sterile alpha motif, type 1||Serine/threonine/dual specificity protein kinase, catalytic domain||Serine-threonine/tyrosine-protein kinase catalytic domain||Sterile alpha motif, type 2||Tyrosine-protein kinase, catalytic domain||Protein kinase-like domain||Sterile alpha motif domain||Fibronectin type III||Sterile alpha motif/pointed domain||Galactose-binding domain-like||Protein kinase domain||Tyrosine-protein kinase ephrin type A/B receptor-like
ENSGALG00000000569	90.5738298	44.49747632	1.0254	0.015522	SDC3	Syndecan/Neurexin domain||Neurexin/syndecan/glycophorin C
ENSGALG00000003578	946.1660244	469.732274	1.0103	0.011789	fn1	Fibronectin, type II, collagen-binding||Fibronectin type III||Fibronectin, type I||Kringle-like fold
ENSGALG00000010625	241.7713737	120.5590431	1.0039	0.029456	SRD5A2	Protein of unknown function DUF1295||3-oxo-5-alpha-steroid 4-dehydrogenase||3-oxo-5-alpha-steroid 4-dehydrogenase, C-terminal
ENSGALG00000026047	52.34292792	26.18910852	0.99903	0.04913	AEBP1	Carboxypeptidase-like, regulatory domain||Peptidase M14, carboxypeptidase A
ENSGALG00000007866	72.43010457	36.35478155	0.99444	0.046266	BCL11A	Zinc finger, C2H2||Zinc finger, C2H2-like
ENSGALG00000006324	116.9027151	58.97206936	0.9872	0.024233	PFKFB3	Histidine phosphatase superfamily, clade-1||Bifunctional 6-phosphofructo-2-kinase/fructose-2, 6-bisphosphate 2-phosphatase||P-loop containing nucleoside triphosphate hydrolase||Histidine phosphatase superfamily||Fructose-2,6-bisphosphatase||6-phosphofructo-2-kinase
ENSGALG00000002532	28.8473482	14.56987832	0.98545	0.03516	GJA4	Connexin||Connexin, N-terminal||Gap junction protein, cysteine-rich domain
ENSGALG00000017417	28.21689327	14.26655986	0.98392	0.04553	USP43	Ubiquitin specific protease domain||Peptidase C19, ubiquitin carboxyl-terminal hydrolase
ENSGALG00000028291	581.9409236	296.1471476	0.97456	0.024752	BCAR1	Serine rich protein interaction||Spectrin alpha chain, SH3 domain||Variant SH3 domain||CAS family, DUF3513||SH3 domain
ENSGALG00000009732	48.27633874	24.60708528	0.97224	0.040791	PCDH18	Cadherin, N-terminal||Cadherin||Cadherin-like
Novel00584	270.3332075	137.8891597	0.97123	0.02074	-//-	
ENSGALG00000006130	37.00004353	18.94680776	0.96557	0.036366	STMN3	Stathmin family
ENSGALG00000002667	60.05541863	30.77453746	0.96456	0.0359	OLFML2B	Olfactomedin-like||Nucleic acid-binding, OB-fold
ENSGALG00000017308	24.49076543	12.56803147	0.96248	0.043419	CHRDL2	von Willebrand factor, type C
ENSGALG00000006883	227.2230198	116.668131	0.9617	0.032058	ENTHD2	Armadillo-type fold||ENTH/VHS||ENTH domain
ENSGALG00000007357	262.1422627	134.8004441	0.95952	0.015092	BCL6	Zinc finger, C2H2||BTB/POZ||BTB/POZ fold||BTB/POZ-like||Zinc finger, C2H2-like
ENSGALG00000026663	21.3859024	11.02446201	0.95595	0.048355	CX3CL1	Chemokine interleukin-8-like domain
ENSGALG00000003103	166.7555893	86.18662402	0.9522	0.018516	MST1R	Protein kinase domain||Tyrosine-protein kinase, HGF/MSP receptor||Sema domain||Protein kinase-like domain||Serine-threonine/tyrosine-protein kinase catalytic domain||Plexin-like fold||IPT domain||Tyrosine-protein kinase, catalytic domain||Immunoglobulin E-set||Serine/threonine/dual specificity protein kinase, catalytic domain
ENSGALG00000016988	133.1799817	68.90243514	0.95075	0.046642	KIAA0226L	-
Novel00918	97.1916712	50.36537002	0.9484	0.037332	-//-	
ENSGALG00000012023	59.46882676	30.90935453	0.94409	0.022809	DACT1	-
ENSGALG00000026957	283.2590186	149.3531903	0.92339	0.026698	SEMA4G	Plexin-like fold||Plexin||Immunoglobulin subtype||Sema domain
ENSGALG00000002955	52.24016223	27.61792077	0.91955	0.042731	ADAMTSL2	Thrombospondin, type 1 repeat||ADAM-TS Spacer 1||Peptidase M12B, ADAM-TS||PLAC
ENSGALG00000001478	38.19655069	20.25431749	0.91521	0.042612	-	Zinc finger, RING-type||L-Aspartase-like||Protein of unknown function DUF3583||Zinc finger, B-box||Ribonuclease H-like domain
ENSGALG00000026450	365.7625577	194.1590712	0.91367	0.02106	PLEKHG3	Pleckstrin homology domain||Dbl homology (DH) domain
ENSGALG00000023094	315.3776987	167.4351201	0.91348	0.021657	-//-	
ENSGALG00000013502	193.7513897	103.7874254	0.90057	0.046647	GCC1	GRIP
ENSGALG00000003923	281.6425866	150.9137062	0.90014	0.018594	COL6A3	Collagen triple helix repeat||von Willebrand factor, type A||Proteinase inhibitor I2, Kunitz metazoa||Fibronectin type III
Novel00639	93.50652148	50.2529328	0.89586	0.032473	-//-	
ENSGALG00000007807	577.4817422	310.6803114	0.89434	0.038752	VASN	Fibronectin type III||Epidermal growth factor-like domain||Leucine-rich repeat||Leucine-rich repeat, typical subtype||Cysteine-rich flanking region, C-terminal
ENSGALG00000015926	81.1791214	43.73449447	0.89234	0.035345	RUNX1T1	Eight-Twenty-One||TAFH/NHR1||Myeloid transforming gene on chromosome 8 (MTG8)||NHR2-like||Zinc finger, MYND-type
ENSGALG00000024318	42.24152259	22.76426744	0.89189	0.044852	TMEM200B	Protein of unknown function DUF2371, TMEM200
ENSGALG00000003577	138.0052711	74.79260935	0.88376	0.033684	RNF19B	Zinc finger, C6HC-type||Zinc finger, RING-type
ENSGALG00000026718	188.5605064	102.5031226	0.87936	0.023898	HIC1	BTB/POZ-like||Zinc finger, C2H2-like||Zinc finger, C2H2||BTB/POZ||BTB/POZ fold
ENSGALG00000008297	625.2775739	340.4565885	0.87703	0.02323	SEMA4B	Sema domain||Immunoglobulin-like domain||Plexin||Plexin-like fold
ENSGALG00000014795	345.0562313	188.3454662	0.87345	0.022674	TGIF1	Homeobox KN domain||Homeodomain-like||Homeobox domain
ENSGALG00000028531	190.0401067	103.9857365	0.86992	0.028203	-	Pleckstrin homology domain
ENSGALG00000004893	64.49013116	35.47375723	0.86233	0.039677	TMEM119	-
ENSGALG00000007063	307.7647842	169.3983877	0.86141	0.027877	CBX4	-
ENSGALG00000008947	98.61840688	55.09584491	0.83991	0.046409	PAK7	Serine/threonine/dual specificity protein kinase, catalytic domain||Protein kinase domain||Protein kinase-like domain||CRIB domain||Serine-threonine/tyrosine-protein kinase catalytic domain||Tyrosine-protein kinase, catalytic domain
ENSGALG00000004673	78.54403321	44.19717854	0.82955	0.042889	C10orf54	Immunoglobulin V-set domain||Immunoglobulin subtype||Immunoglobulin-like domain
ENSGALG00000006681	190.2813862	108.0313079	0.81668	0.02279	BRSK2	Protein kinase-like domain||Protein kinase domain||Tyrosine-protein kinase, catalytic domain||Serine-threonine/tyrosine-protein kinase catalytic domain||Serine/threonine/dual specificity protein kinase, catalytic domain
ENSGALG00000004662	57.98003952	33.11776678	0.80795	0.049067	DISP3	Sterol-sensing domain||Membrane transport protein, MMPL domain||Patched
ENSGALG00000005768	217.7264342	126.2088609	0.7867	0.049033	ETV5	Ets domain||PEA3-type ETS-domain transcription factor, N-terminal
ENSGALG00000017414	759.2142132	446.9393281	0.76443	0.038731	JUP	Armadillo-type fold||Armadillo||Beta-catenin
ENSGALG00000014554	379.005796	223.3013702	0.76323	0.04598	ATN1	Atrophin-like||Atrophin-1
ENSGALG00000027630	710.5297567	423.4165153	0.74682	0.046237	NPR2	Extracellular ligand-binding receptor||Protein kinase domain||Natriuretic peptide receptor||Serine/threonine/dual specificity protein kinase, catalytic domain||Periplasmic binding protein-like I||Adenylyl cyclase class-3/4/guanylyl cyclase||Nucleotide cyclase||Protein kinase-like domain||Serine-threonine/tyrosine-protein kinase catalytic domain||Tyrosine-protein kinase, catalytic domain
ENSGALG00000001717	659.0357882	395.685088	0.736	0.048853	SLC16A1	Monocarboxylate transporter||Major facilitator superfamily||Major facilitator superfamily domain
ENSGALG00000013722	104.9558206	64.08849201	0.71164	0.046229	SEMA3B	Sema domain
Novel00230	163.5416224	277.203206	-0.76129	0.03926	--	PREDICTED: transmembrane channel-like 5 isoform X10 [Gallus gallus]>gi|513208725|ref|XP_414913.4| PREDICTED: transmembrane channel-like 5 isoform X9 [Gallus gallus]
Novel00631	931.9944713	1601.148241	-0.78071	0.045887	-//-	
ENSGALG00000015071	134.5431054	233.1149403	-0.79297	0.04235	FOCAD	Armadillo-type fold||Domain of unknown function DUF3730||Protein of unknown function DUF3028
ENSGALG00000009847	141.8272175	248.6631218	-0.81006	0.035026	STXBP6	Synaptobrevin
ENSGALG00000006193	248.2038977	438.3377584	-0.82052	0.038106	TXNRD3	Pyridine nucleotide-disulphide oxidoreductase, NAD-binding domain||Glutaredoxin||FAD/NAD-linked reductase, dimerisation domain||FAD binding domain||Pyridine nucleotide-disulphide oxidoreductase, FAD/NAD(P)-binding domain||Pyridine nucleotide-disulphide oxidoreductase, dimerisation domain||Glutaredoxin subgroup||FAD-dependent pyridine nucleotide-disulphide oxidoreductase||Thioredoxin/glutathione reductase selenoprotein||Mercuric reductase||Glutaredoxin, eukaryotic/virial||Thioredoxin-like fold
ENSGALG00000008076	140.3493175	248.2446781	-0.82274	0.038122	TMEM164	-
ENSGALG00000016502	126.2949039	225.697858	-0.8376	0.03396	TP53I3	GroES (chaperonin 10)-like||Polyketide synthase, enoylreductase domain||Alcohol dehydrogenase GroES-like||Quinone oxidoreductase PIG3||Alcohol dehydrogenase, C-terminal
ENSGALG00000002152	818.0543911	1461.963984	-0.83764	0.027058	GABRP	Gamma-aminobutyric acid A receptor/Glycine receptor alpha||Gamma-aminobutyric acid A receptor pi subunit||Neurotransmitter-gated ion-channel ligand-binding domain||Neurotransmitter-gated ion-channel transmembrane domain||Neurotransmitter-gated ion-channel
ENSGALG00000006976	45.66401755	82.3735404	-0.85112	0.037578	-	Glucose/ribitol dehydrogenase||Short-chain dehydrogenase/reductase SDR||Polyketide synthase, ketoreductase domain
ENSGALG00000007892	94.50270074	173.7104024	-0.87826	0.041584	SPAG6	Armadillo-type fold||26S proteasome non-ATPase regulatory subunit 5||Armadillo
ENSGALG00000002021	85.40491161	157.4907255	-0.88288	0.038058	ZC3H12A	Ribonuclease Zc3h12a-like
ENSGALG00000012801	250.5196867	464.3984309	-0.89044	0.015797	LY86	MD-2-related lipid-recognition domain||Immunoglobulin E-set
ENSGALG00000003176	23.13980336	42.95174343	-0.89234	0.048307	-	Rho GTPase-activating protein domain||Rho GTPase activation protein
ENSGALG00000015787	32.83566569	61.34835382	-0.90176	0.042369	RNGTT	Dual specificity phosphatase, catalytic domain||mRNA capping enzyme, bifunctional||Nucleic acid-binding, OB-fold||mRNA capping enzyme||DNA ligase, ATP-dependent, central||Protein-tyrosine phosphatase, receptor/non-receptor type||Protein-tyrosine phosphatase-like||Protein-tyrosine/Dual specificity phosphatase||mRNA capping enzyme, C-terminal
ENSGALG00000014127	23.53772375	44.03890846	-0.9038	0.032922	TXK	Protein kinase-like domain||Protein kinase domain||SH3 domain||Serine-threonine/tyrosine-protein kinase catalytic domain||Tyrosine-protein kinase, catalytic domain||Serine/threonine/dual specificity protein kinase, catalytic domain||SH2 domain
ENSGALG00000004559	24.48809193	45.86686181	-0.90537	0.034847	DPY19L3	Dpy-19/Dpy-19-like
ENSGALG00000002347	47.95655344	90.02123258	-0.90854	0.037324	C3orf58	-
ENSGALG00000016212	15.02500038	28.26946765	-0.91188	0.046498	LICOS	Immunoglobulin V-set domain||Immunoglobulin C1-set||Immunoglobulin subtype||Immunoglobulin-like domain||CD80-like, immunoglobulin C2-set
ENSGALG00000003550	122.1288948	230.2580722	-0.91485	0.0268	ARHGAP40	Rho GTPase-activating protein domain||Rho GTPase activation protein
ENSGALG00000002504	83.06424538	158.0276169	-0.92788	0.022502	TROVE2	von Willebrand factor, type A||TROVE
ENSGALG00000017197	12.76412933	24.3198431	-0.93004	0.047073	FAR-2	Immunoglobulin||Immunoglobulin V-set domain||Immunoglobulin subtype 2||Immunoglobulin I-set||Fibronectin type III||Immunoglobulin-like domain||Immunoglobulin subtype
ENSGALG00000023552	89.97115405	172.4542651	-0.93868	0.0203	SPEF2	P-loop containing nucleoside triphosphate hydrolase||Protein of unknown function DUF1042||Calponin homology domain
ENSGALG00000009936	16.73301955	32.10681567	-0.94018	0.036484	AP4S1	AP complex, mu/sigma subunit||Longin-like domain||Adaptor protein complex, sigma subunit
ENSGALG00000006192	224.1458424	433.143827	-0.95041	0.0066138	USH1C	Mannose-6-phosphate receptor binding domain||PDZ domain
ENSGALG00000004684	105.1241204	203.8809237	-0.95563	0.022968	IPPK	Inositol-pentakisphosphate 2-kinase
ENSGALG00000015503	114.9397503	223.767735	-0.96112	0.032615	GGH	Peptidase C26||Glutamine amidotransferase||Class I glutamine amidotransferase-like
ENSGALG00000014072	93.2044932	184.8174345	-0.98763	0.015177	TSPAN18	Tetraspanin/Peripherin||Tetraspanin||Tetraspanin, EC2 domain
ENSGALG00000015554	18.75950939	37.88613926	-1.014	0.031912	-	VPS10||PKD domain
ENSGALG00000006095	17.65687739	35.84349709	-1.0215	0.047542	-	FERM central domain||Protein kinase-like domain||FERM domain||FERM, N-terminal||FERM, C-terminal PH-like domain||Band 4.1 domain||Ubiquitin-related domain||KIND
ENSGALG00000010187	19.37738339	39.41065223	-1.0242	0.046564	SLC1A1	Sodium:dicarboxylate symporter
ENSGALG00000006889	227.0231224	474.5687282	-1.0638	0.010955	TMC7	TMC
ENSGALG00000009615	28.30008652	59.38604559	-1.0693	0.034131	RRP15	Protein of unknown function DUF1665
Novel00571	1403.13221	2953.128256	-1.0736	0.018561	--	OvoDB1 [Taeniopygia guttata]
ENSGALG00000005347	54.5578589	116.6911047	-1.0968	0.046984	ADAMTS18	PLAC||Peptidase M12B, ADAM-TS||Thrombospondin, type 1 repeat||ADAM-TS Spacer 1||Peptidase M12B, ADAM/reprolysin||Peptidase M12B, propeptide
ENSGALG00000015940	34.56529541	75.38493999	-1.125	0.021213	TMEM67	Meckelin||Insulin-like growth factor binding protein, N-terminal
ENSGALG00000011099	12.60398337	27.61802654	-1.1317	0.037911	FAP	Alpha/Beta hydrolase fold||Peptidase S9, prolyl oligopeptidase, catalytic domain||Peptidase S9B, dipeptidylpeptidase IV N-terminal
ENSGALG00000016161	7.192068289	16.25845743	-1.1767	0.031647	TSNARE1	Syntaxin, N-terminal domain||Target SNARE coiled-coil domain||t-SNARE
ENSGALG00000001841	51.40641991	117.1070458	-1.1878	0.0043154	ROGDI	RAVE subunit 2/Rogdi
ENSGALG00000002242	14.00622612	31.99245981	-1.1917	0.024983	GALNT9	Nucleotide-diphospho-sugar transferases||Glycosyltransferase 2-like||Ricin B lectin domain
ENSGALG00000015126	9.814283349	22.50532293	-1.1973	0.019137	TRPM3	Ion transport domain
ENSGALG00000014712	23.26121996	53.4042124	-1.199	0.0053381	SLC38A9	Amino acid transporter, transmembrane
ENSGALG00000011225	12.4596864	29.33604329	-1.2354	0.014627	SLC25A47	Mitochondrial carrier protein||Mitochondrial substrate/solute carrier||Mitochondrial carrier domain
Novel00112	35.22551539	84.3787997	-1.2603	0.0020215	-//-	
ENSGALG00000017268	8.820581137	21.31333373	-1.2728	0.012318	KCTD21	BTB/POZ-like||Potassium channel tetramerisation-type BTB domain||BTB/POZ fold
ENSGALG00000019283	58.3438579	143.6243053	-1.2996	0.034229	-	Aldo/keto reductase subgroup||NADP-dependent oxidoreductase domain
ENSGALG00000012486	4.820374232	11.9080417	-1.3047	0.047646	KCTD17	BTB/POZ fold||Potassium channel tetramerisation-type BTB domain||BTB/POZ-like
ENSGALG00000000845	108.9065098	273.754419	-1.3298	0.034693	UVRAG	C2 domain
Novel00677	3.975635315	10.07745578	-1.3419	0.034136	-//-	
ENSGALG00000013051	26.33985007	66.86935213	-1.3441	0.039585	SEMA5A	Sema domain||Thrombospondin, type 1 repeat||Plexin-like fold
Novel00175	45.42654079	118.4324158	-1.3825	0.00050426	--	PREDICTED: fatty acyl-CoA hydrolase precursor, medium chain-like isoform X4 [Gallus gallus]
ENSGALG00000027244	6.617437706	17.78726958	-1.4265	0.018899	-	Glucosamine/galactosamine-6-phosphate isomerase||Glucosamine-6-phosphate isomerase
ENSGALG00000012236	5.002369193	13.68192509	-1.4516	0.022001	DMC1	Circadian clock protein KaiC/DNA repair protein RadA||Meiotic recombinase Dmc1||DNA recombination and repair protein, RecA-like||DNA repair Rad51/transcription factor NusA, alpha-helical||AAA+ ATPase domain||DNA recombination and repair protein Rad51, C-terminal||DNA recombination and repair protein RecA||DNA recombination and repair protein RecA, monomer-monomer interface||DNA recombination and repair protein RecA-like, ATP-binding domain||P-loop containing nucleoside triphosphate hydrolas
ENSGALG00000015360	6.63942893	18.60217707	-1.4863	0.011018	KIAA1524	Armadillo-type fold
ENSGALG00000023724	6.922861642	19.83481877	-1.5186	0.0061414	SNX20	Phox homologous domain
ENSGALG00000022718	3.286251508	9.630817651	-1.5512	0.032284	FUT4	Glycosyl transferase, family 10
ENSGALG00000014406	4.310954627	12.74495938	-1.5638	0.013996	PACRGL	Armadillo-type fold||Parkin co-regulated protein
Novel00529	2.562037163	7.679925949	-1.5838	0.048687	-//-	
ENSGALG00000014903	14.03922898	43.80350066	-1.6416	0.018575	ITGA2	FG-GAP repeat||Integrin alpha-2||Integrin alpha chain||von Willebrand factor, type A||Integrin alpha beta-propellor
ENSGALG00000027693	6.330711153	20.99693902	-1.7297	0.022002	-	Immunoglobulin C1-set||Immunoglobulin-like domain||MHC classes I/II-like antigen recognition protein||MHC class I alpha chain, alpha1 alpha2 domains||MHC class I alpha chain
ENSGALG00000002109	1.480477725	5.163265739	-1.8022	0.041797	KCTD19	Potassium channel tetramerisation-type BTB domain||BTB/POZ fold
ENSGALG00000016227	3.36787301	12.01125344	-1.8345	0.047669	GPR34	GPCR, rhodopsin-like, 7TM||G protein-coupled receptor, rhodopsin-like
ENSGALG00000010929	2.947394083	12.32776508	-2.0644	0.013101	SPARCL1	Kazal domain||SPARC-like protein 1||Follistatin/Osteonectin EGF domain||EF-hand domain||Follistatin-like, N-terminal||SPARC/Testican, calcium-binding domain
ENSGALG00000013766	4.999163023	21.91555185	-2.1322	0.0022729	DSEL	Chondroitin AC/alginate lyase||P-loop containing nucleoside triphosphate hydrolase||Sulfotransferase domain
ENSGALG00000026625	31.81723025	201.015989	-2.6594	0.00032005	-//-	
ENSGALG00000026075	1.89153255	12.31725351	-2.7031	0.017088	AMER3	Uncharacterised protein family FAM123
ENSGALG00000011933	1.401182787	10.68814729	-2.9313	0.012702	-	Armadillo-type fold
ENSGALG00000011041	0.772489217	7.766455174	-3.3297	0.011446	DNAJC6	DnaJ domain||Protein-tyrosine/Dual specificity phosphatase||Protein-tyrosine phosphatase-like||C2 domain||Tensin phosphatase, lipid phosphatase domain||Tensin phosphatase, C2 domain
ENSGALG00000003446	96.8719636	1125.405476	-3.5382	8.14E-06	PRLR	Fibronectin type III||Growth hormone/erythropoietin receptor, ligand binding||Interleukin-6 receptor alpha, binding
ENSGALG00000025433	0.148699819	3.546769476	-4.576	0.0107	-//-	
